# Recruitment and Retention in Diverse Cohorts: Lessons From Community-Engaged Efforts

**DOI:** 10.1093/geroni/igab046.1821

**Published:** 2021-12-17

**Authors:** Carrie Nieman, Haera Han, George Rebok

## Abstract

Effective behavioral interventions and associated trials reflect the complexity and context of the communities with which they are tailored and the behaviors they seek to address. Community-engaged methodology can serve to capture these complexities, particularly when focusing on health inequities. Significant health and healthcare disparities persist among racial/ethnic minorities and representation of racial/ethnic minorities is lacking within trials that reflects the diversity of the U.S. population. Novel approaches are needed to increase the diversity of participants within behavioral intervention research. This symposium covers the unique barriers and facilitators related to recruitment and retention across a range of populations, including African American and Hispanic/LatinX older adults with hearing loss to diverse dementia family caregivers and community-dwelling Korean American older adults. Beyond the challenges and opportunities, the symposium will focus on effective recruitment strategies. The discussion will include 1) findings from 10 years of recruiting older Korean Americans into community-based trials, 2) lessons in tailoring recruitment efforts to dementia family caregivers, 3) the integration of human-centered design into a community-engaged hearing care intervention targeting low-income and African American older adults, 4) successful recruitment and retention efforts in a community-based participatory research trial in a borderlands community, and 5) the deployment of strategies to recruit Latino, Asian, and African American older adults with depression and anxiety in the setting of the COVID-19 pandemic. This symposium seeks to build the evidence related to recruitment of older racial/ethnic minorities in diverse settings, which is fundamental to addressing health inequities through behavioral intervention research.

